# P-189. Impact of Malaria Preventive Measures on U.S. Military Operations in the Pacific: A Historical and Modern Perspective

**DOI:** 10.1093/ofid/ofaf695.412

**Published:** 2026-01-11

**Authors:** Derek Thomas Larson, Nanda Ramchandar

**Affiliations:** US Navy, San Diego, CA; Naval Medical Center San Diego, San Diego, California

## Abstract

**Background:**

Malaria has had a persistent and significant impact on the development of human history and the wars waged throughout it. The impacts on United Stated military operations have been documented since the Revolutionary War in 1776, when one of the first purchases of the Continental Congress was a supply of quinine to protect George Washington’s troops. Modern conflicts, including the World Wars, the Korean War, and the Vietnam war, have had a more thorough accounting of the disruption of malaria. Publications from these wars have reported the attack rate of malaria, servicemember duty days lost, and overall attributable mortality. Robust literature also exists on the effectiveness of chemoprophylaxis for malaria, which during active conflict may be the only utilizable method of prevention. Further, adherence to these preventative measures has been shown to be highly variable amongst US troops. We aim to provide an analysis of the potential impact of malaria on military operations in the Indo-pacific region on both human life and cost.

**Figure:**
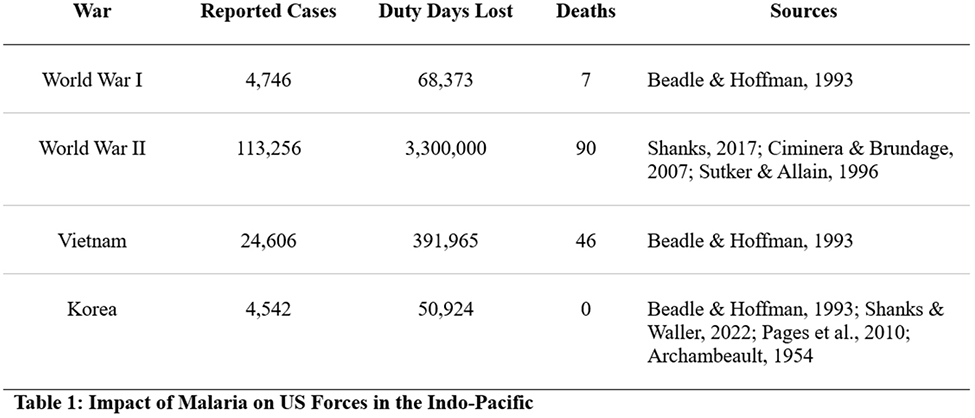

**Figure:**
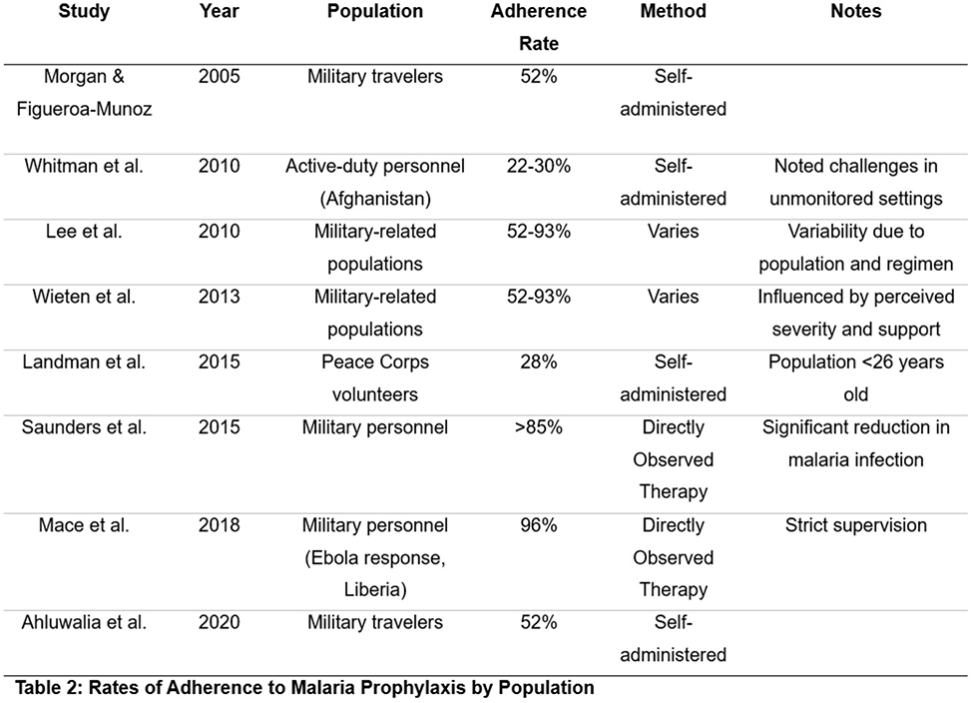

**Methods:**

Indexed literature from PubMed was reviewed relevant to the World Wars, the Korean War, and the Vietnam War as well as the rates of self-adherence to malaria prophylaxis in servicemembers and traveling civilians. We then extrapolated that data to look at morbidity and mortality per 1000 persons at risk and the mitigating effect of leadership (Table 3).

**Figure:**
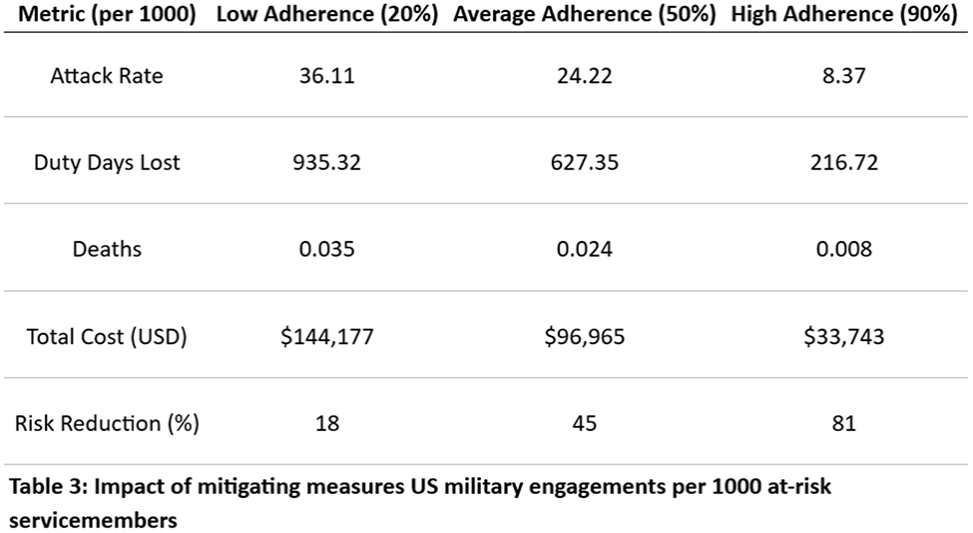

**Results:**

15 relevant publications were included in our analysis. The efficacy of malaria prophylaxis was consistently reported to be 90-100% when adherence was high. Estimated incidence and aggregate duty days lost are shown in Table 1. Adherence to prophylaxis measures varied widely from 20% to over 90% in some studies (Table 2). Our analysis demonstrated an over 70% estimated reduction in the attack rate with high adherence to prophylaxis (Table 3).

**Conclusion:**

Malaria mitigation strategies are highly effective when adherence is high and significantly decrease the observed disease attack rate, improve operational readiness, and decrease troop morbidity and mortality.

**Disclosures:**

All Authors: No reported disclosures

